# Functional and radiological outcomes after treatment with custom-made acetabular components in patients with Paprosky type 3 acetabular defects: short-term results

**DOI:** 10.1186/s12891-020-03851-9

**Published:** 2020-12-10

**Authors:** Michael S. Gruber, Michael Jesenko, Julia Burghuber, Josef Hochreiter, Peter Ritschl, Reinhold Ortmaier

**Affiliations:** 1 Unfallkrankenhaus Linz, Linz, 4020 Upper Austria Austria; 2 Orthopedic Hospital Gersthof, Vienna, 1180 Austria; 3 Department of Orthopedic Surgery, Ordensklinikum Barmherzige Schwestern Linz, Vinzenzgruppe Center of Orthopedic Excellence, Teaching Hospital of the Paracelsus Medical University Salzburg, Linz, 4020 Austria; 4 Institute for Sports Medicine, Alpine Medicine and Health Tourism (ISAG), Tirol Kliniken GmbH, Innsbruck and UMIT, Hall Austria, Innsbruck, 6020 Tyrol Austria

**Keywords:** CTAC, 3D-printed prosthesis, Revision surgery, Severe acetabular bone loss, Custom-made acetabular component, Total hip arthroplasty

## Abstract

**Background:**

Severe acetabular defects require special treatment with either impaction bone grafting, metal augmented cups or cup-cage constructs. Even these options are often not adequate, especially in hips with Paprosky type 3 defects with loss of anterior and posterior columns. This study investigates the clinical and radiological outcomes of custom-made acetabular components (© Materialise NV, Leuven, Belgium) for Paprosky type 3 defects.

**Methods:**

Sixteen patients were eligible for this trial, nine of whom agreed to be included. All of them completed one year of follow-up. The Harris hip score and the Oxford hip score were used to compare pre- and postoperative functional outcomes. Radiological follow-up comprised anteversion and inclination of the implanted cup and offset measurements in both hips (femoral, medial, ischial offset and center of rotation). Statistical analyses were performed with IBM SPSS Statistics.

**Results:**

The mean follow-up time of the nine patients was 12.2 months (range: 10–18). The Oxford hip score and Harris hip score improved from 19.8 and 50.1 to 29.4 and 68.8, respectively (*p* = 0.009 and 0.01). There were complications in three cases (33.3%), which led to one re-revision (11.1%). Radiologic follow-up showed restoration of the height of the center of rotation and of the global offset. Significant difference was detected in the femoral offset.

**Conclusions:**

The functional and radiological outcomes are promising. However, long-term outcomes still need to be examined.

**Level of evidence:**

Therapeutic Level IV.

## Background

The number of total hip arthroplasties (THAs) has increased in recent decades, and a further increase in incidence is predicted [1–3]. The reasons for this increase are multifactorial and comprise an increasing incidence of THA in younger as well as in older [3].

However, with an increasing number of primary THAs, the absolute number of revisions will automatically increase, and orthopedic surgeons will be increasingly forced to deal with revision surgeries.

Reasons for revisions are dislocation/instability (22%), mechanical loosening (20%), infection (15%) and implant failure (10%) [4]. In terms of revision, extensive acetabular defects are one of the most challenging situations for surgeons. While many contained defects can be managed with the use of standard cups, uncontained extensive defects require other strategies. Acetabular defects can be approached mainly with three methods, namely, impaction bone grafting (IBG), metal augmented cups or cup-cage constructs [5].

IBG was first described by Hastings and Parker in 1975 [6], and initial reports showed high overall failure rates. Later, the Exeter and Nijmegen groups reported improved outcome with IBG and the usage of cemented cups [7, 8]. The results also improved thanks to the use of cementless, porous-coated cups, which lead to improve bone integration [9, 10]. However, this technique is limited by the severity of the defect, and the outcome is linked to the Paprosky type of defect [11].

Metal augments have been used for many years now. Tantalum is the material of choice. It can be shaped in a highly porous “foam”, with low stiffness and high friction. These implants can be placed directly at the area of the defect and can restore the center of rotation (COR). However, as in IBG, metal-augmented implants need a certain amount of host bone to achieve sufficient fixation for integration [12].

For more severe defects or pelvic discontinuities, cup-cage constructs are available. With the use of these stiff implants, Martin et al. reported relatively good outcomes and survivorship [13].

However, in regard to combined defects with anterior and posterior column deficiency, established implants do not provide satisfactory results in terms of COR position [14, 15].

As an alternative to the abovementioned methods, newly and specially designed custom-made triflanged acetabular components (CTACs) are on the rise. These can be produced and adapted for each unique acetabular defect based on preoperative CT images. The main goals of revision surgery for acetabular defects are to restore the biomechanics of the hip (COR), to achieve implant stability and to restore bone stock [16]. CTACs were developed to achieve implant stability and restore hip biomechanics in cases with severe bone loss. Although this method is quite expensive compared to the use of standard implants [17, 18], it may often be the only possible solution for THA revision.

The purpose of this retrospective cohort study was to evaluate the clinical and radiological results after treatment with CTAC cups for revision surgery in patients with severe Paprosky 3A and 3B acetabular defects.

## Methods

In the period from April 2016 to April 2018, 16 patients received treatment with aMace (© Materialise NV, Leuven, Belgium) CTACs for revision THA with concomitant acetabular type 3A or 3B bone defects (Table 1). Of these sixteen patients, nine agreed to be included and followed up in this trial. Four patients were treated at the hospital “Ordensklinikum Linz GmbH Barmherzige Schwestern”, and five patients were treated at the Orthopedic Hospital Gersthof. The reasons for revision surgery varied: seven patients received treatment because of aseptic loosening, and one patient each received treatment because of periprosthetic acetabular fracture and septic loosening. Before implantation, infection was excluded through preoperative intraarticular joint aspiration and evaluations of C-reactive protein (CRP) and white blood cell (WBC) count as well as clinical investigations. Additional intraoperative sonication of the dismantled implant and/or examination of five tissue samples verified correct preoperative diagnostics. Three of the nine surgeries were performed on the left hip and six on the right hip. All of the patients included were female, and the patients’ age at the time of surgery was between 42 and 85 years (mean 69.3). The mean body mass index was 29.2 kg/m^2^ (range: 19.8–42.1) (Table 2).

**Table 1:** Paprosky classification of acetabular bone loss – created according to Classifications In Brief - Paprosky Classification of Acetabular Bone Loss [19]

Defect	Tear drop	Superior dome	Anterior column	Posterior column	Bone bed
Type 1	Present	No migration	Intact	Intact	Mild (> 50% cancellous)
Type 2A	Intact	Mild migration < 2 cm superior	Intact	Intact	Moderate (< 50% cancellous)
Type 2B	Intact	Mild migration < 2 cm superolateral	Intact	Intact	Moderate (< 50% cancellous)
Type 2C	Moderate destruction	Mild migration < 2 cm medial	Disrupted	Intact	Moderate (< 50% cancellous)
Type 3A	Moderate destruction	Severe migration > 2 cm superolateral	Intact	Moderate lysis	Severe 10–2 o’clock loss (40–70% sclerotic)
Type 3B	Complete obliteration	Severe migration > 2 cm superomedial	Disrupted	Severe lysis	Severe 9–5 o’clock loss (30% sclerotic)

**Table 2:** Patient demographics of all patients available for follow-up

	aMace (*n* = 9)	
Age (years/range)	69.3 ± 13.7	
Sex (female/male)	9 (100) / 0 (0)	
Side (left/right)	3 (33.3) / 6 (66.7)	
BMI (kg/m^2^)	29.2 ± 6.9	
Smoker (yes/no)	1 (11.1) / 8 (88.9)	
Paprosky (3A/3B)	1 (11.1) / 8 (88.9)	

Data are presented as the mean ± standard deviation or absolute numbers (percentages)

All patients who agreed to be included into this trial completed radiological and functional follow-up examinations (100%). While clinical follow-up comprised pre- and postoperative evaluation of Oxford hip score (OHS) [20] and Harris hip score (HHS) [21], radiological follow-up included preoperative CT scans and postoperative conventional X-rays. Pre- and postoperative images were compared for implant migration and to determine the correction of the offset. Offset-measurement comprised femoral (femoral axis to center of inlay), medial (center of pelvis to center of inlay) and ilioischial (ilioischial line to center of inlay) offset as well as center of rotation (inter-teardrop line to center of inlay) and was performed with TraumaCad (Brainlab AG, Munich, Germany).

The mean follow-up was 12.2 months (range: 10–18). Radiological investigation showed one patient with Paprosky type 3A and 8 patients with Paprosky type 3B acetabular defects (Table 2). The patients who completed follow-up were hospitalized for a mean of 21.9 days (± 6.3 SD).

Short-term follow-up was chosen due to the limited preexisting evidence and influence of the outcome on the applicability of the implant.

This study was approved by the ethics committee of the hospital “Ordensklinikum Linz GmbH Barmherzige Schwestern” (EKS 25/19). All patients provided informed consent.

### Preoperative evaluation and planning

First, conventional X-rays in 2 planes (anterior-posterior and axial) were performed in the affected hip. Implant positioning and loosening and fractures were documented.

**Fig. 1:**
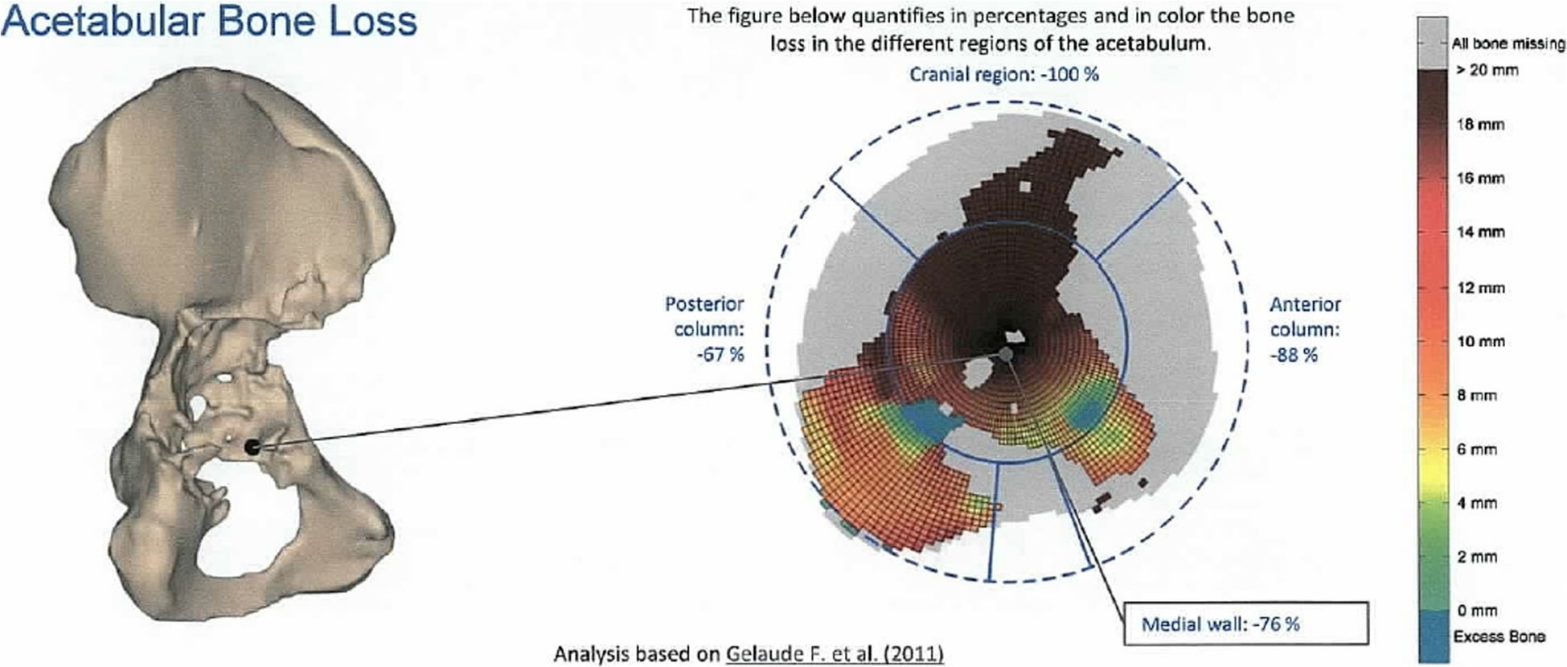
Quantification of acetabular bone loss and assessment of bone quality. Source: planning report provided by Materialise NV, Leuven, Belgium. Permission from the copyright holder to publish the figure has been obtained

**Fig. 2:**
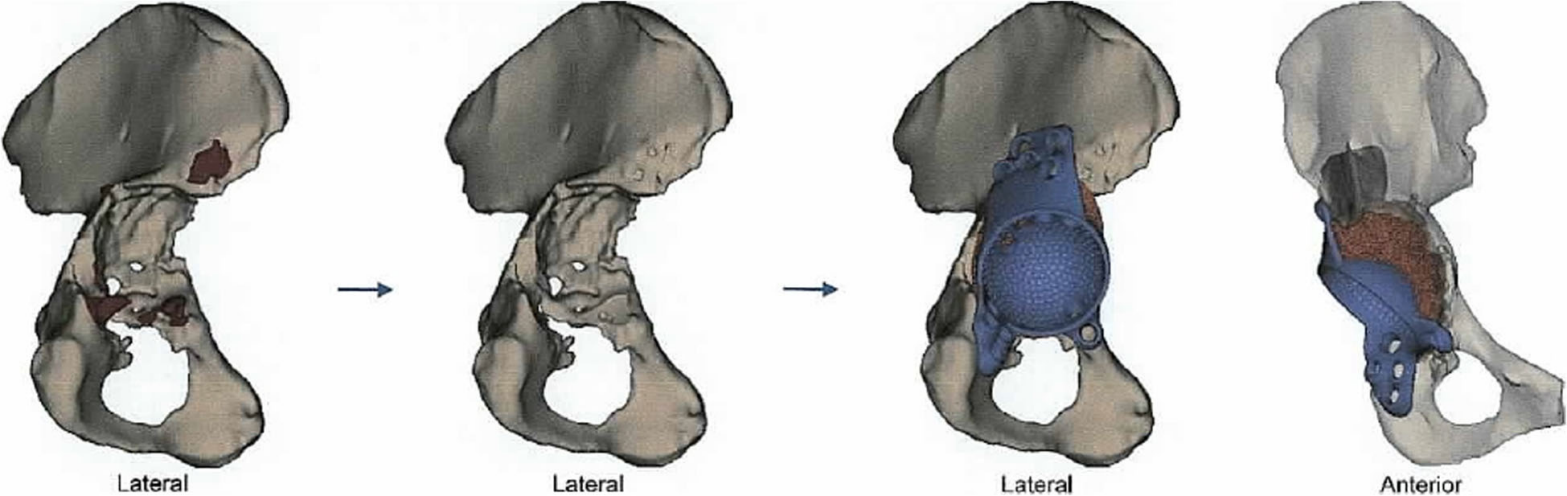
Preparation and reconstruction. From left to right: planned bone removal, bone after removal, proposed implant design (porous augment and plate are built as one part). Source: planning report provided by Materialise NV, Leuven, Belgium. Permission from the copyright holder to publish the figure has been obtained

Then, a CT scan following a special protocol provided by the company was performed. A 3D model of the acetabulum and the pelvis was then virtually conducted, and bone defects and fracture lines were identified. While acetabular bone loss was quantified and classified into the Paprosky score, bone quality was evaluated for fixation of the screws (Figs. 1, 11). An implant design based on the anatomical center of rotation, inclination, anteversion and bone preservation was proposed (Fig. 2). The implant consisted of porous augmentation and the plate, which were built as one part. A titanium alloy was used for the triflanged acetabular cup and the defect filling trabecular augment (Ti6AI4V ELI). Screw length and direction as well as the diameter of the screws were chosen according to bone quality and remaining bone stock.

A preliminary planning report was provided to the surgeon and included instructions for preparation and reconstruction as well as a proposal for screw positioning and length. It was subsequently reviewed by the surgeon and updated to the surgeon’s suggestions via web meetings for improvement. Production started after a final check and confirmation of the final planning report. The listed time for planning and production was 5 weeks (1 and 4 weeks, respectively). The implant came with a trial implant, bone model and custom drill guides. All components were delivered nonsterile and had to be sterilized at the hospital facility. Instruments, screws and cup/liner components were provided by the hospital.

### Surgical technique

Surgery was performed following a standard protocol. Antibiotics were administered preoperatively. Tissue samples for sonication and microbial evaluation were harvested intraoperatively. The procedure was performed in the supine position, and a transgluteal approach was used. For the preparation of proper implant insertion, bone removal and clearance of some bone fragments, according to the planning report, were sometimes necessary, and gaps were filled with morselized allograft bone material afterwards. After bone bed preparation, the trial model of the implant was placed into the acetabulum to confirm implant seating and fitting. Then, the implant was brought in together with a custom drill guide. After drilling, the screw length was confirmed and compared to the planned screw length. After fixation of the acetabular component, a standard or dual mobility cup/liner component was cemented in the implant. The maximum size was predefined by the planning report.

### Postoperative protocol

Patients were mobilized immediately on the first postoperative day after drainage removal. Full weight bearing and full range of motion were allowed immediately after surgery in all patients.

### Statistics

Statistical analyses were performed with IBM SPSS Statistics (Windows, 64 bit, version 23.0; IBM Corp., Armonk, NY, USA). Descriptive statistics were used to investigate patient characteristics. Pre- and postoperative OHS and HHS were compared using paired t-tests. Radiological differences between the treated side of the hip and the contralateral side were evaluated with descriptive statistics and paired t-tests. Statistical significance was reported as a *p*-value of ≤0.05. There were no missing data. Patients who did not complete the follow-up examinations were declared as lost to follow-up and were excluded from the final analysis.

## Results

**Fig. 3:**
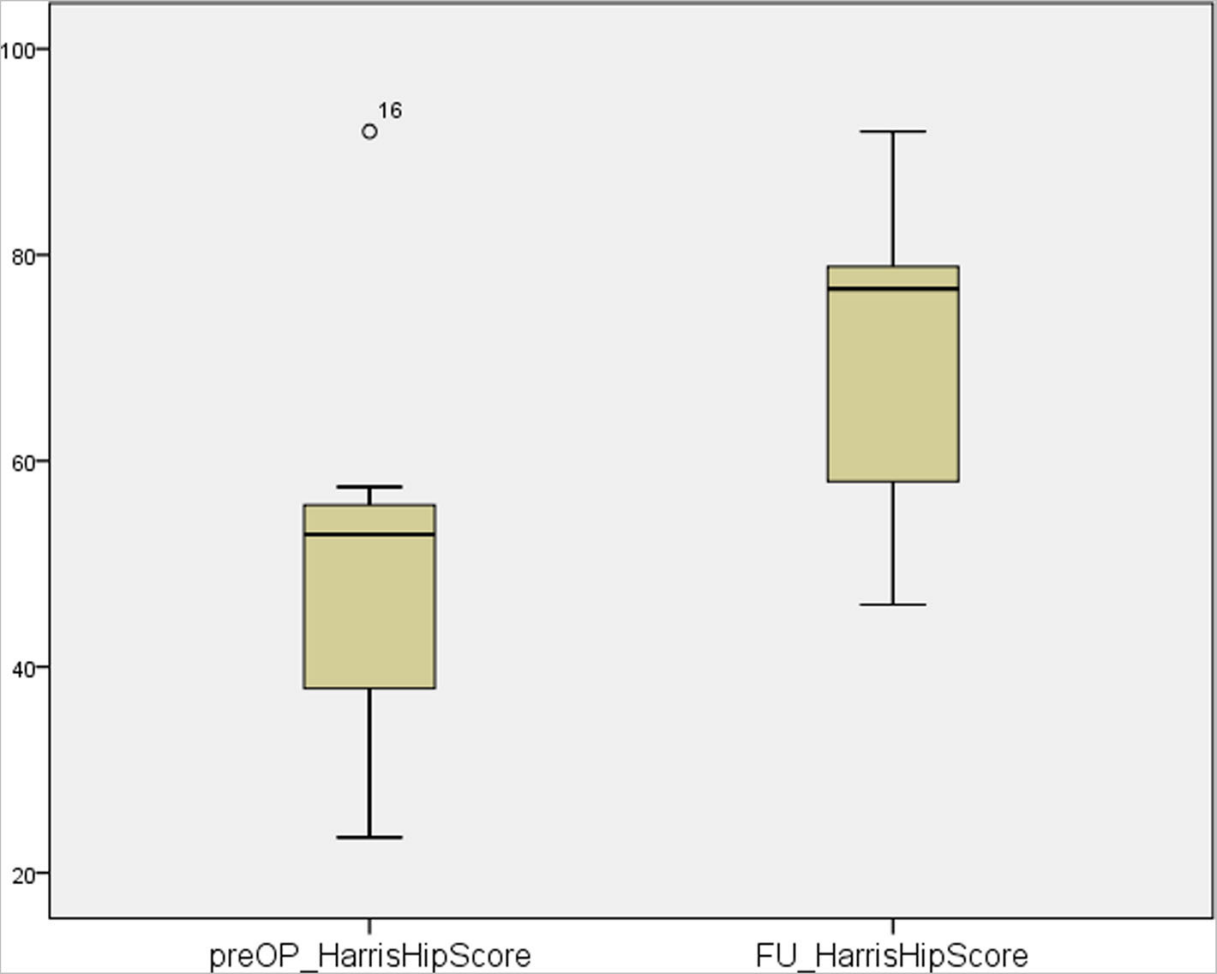
Pre- vs. postoperative Harris Hip Score

**Fig. 4:**
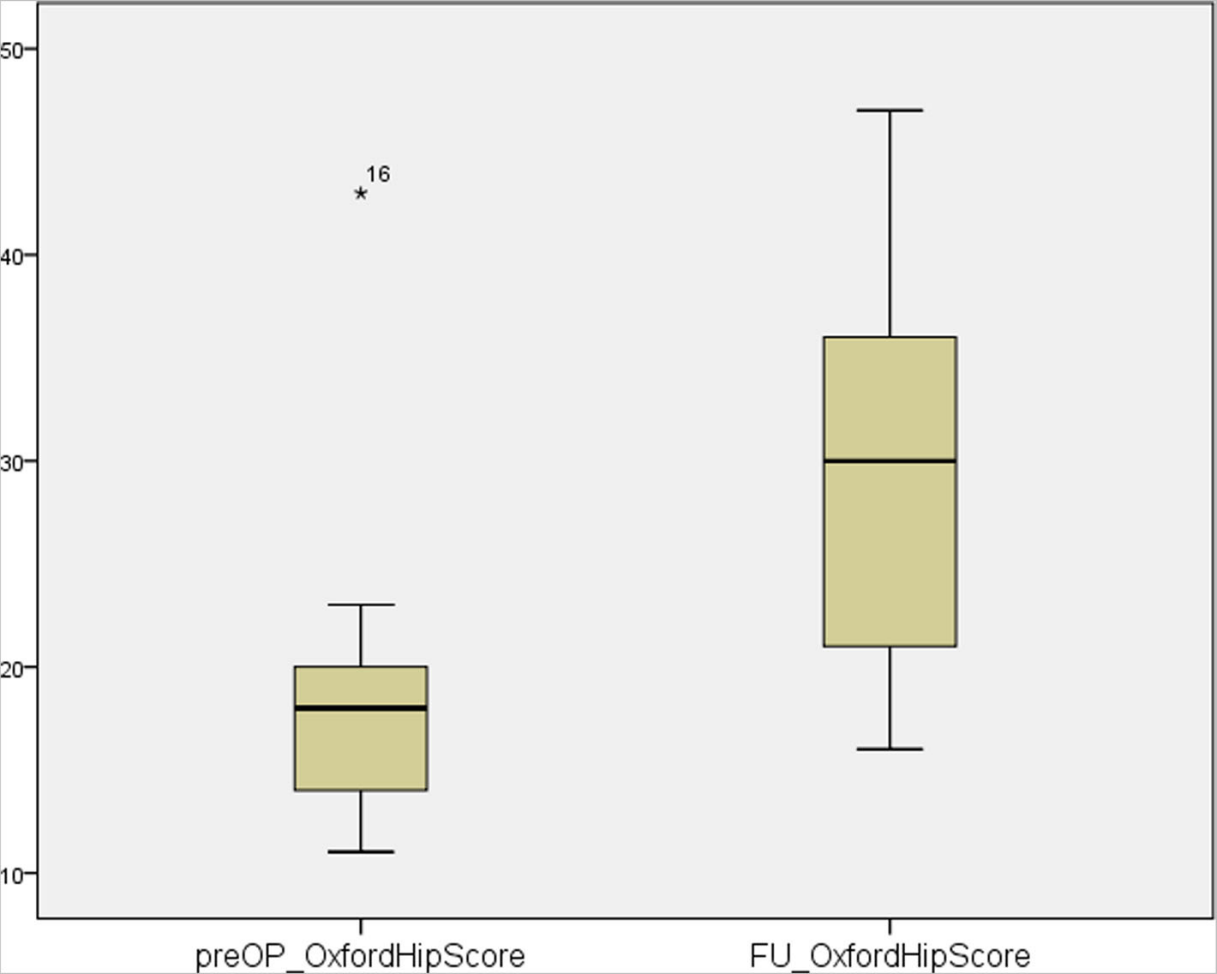
Pre- vs. postoperative Oxford Hip Score

Follow-up results showed an increase in postoperative scores compared to the preoperative assessment. Surgical treatment of patients with severe acetabular bone loss using aMace cups led to significant improvements in the OHS (preoperative median: 18, range: 11–43; postoperative median: 30, range: 16–47; *n* = 9; *p* = 0.009) and HHS (preoperative median: 53, range: 23–92; postoperative median: 77, range: 46–92; n = 9, *p* = 0.01). The patient’s abilities, pain and clinical presentations were significantly better after surgery compared to before. HHS improved in every patient (Fig. 3), whereas 8 out of 9 patients experienced improvements in the OHS (Fig. 4).

During surgery, a mean of 9 screws per patient (range 6–12) were used for fixation of the implant. A mean of 13.7 screws per patient (range 12–16) were planned preoperatively according to the bone condition.

**Fig. 5:**
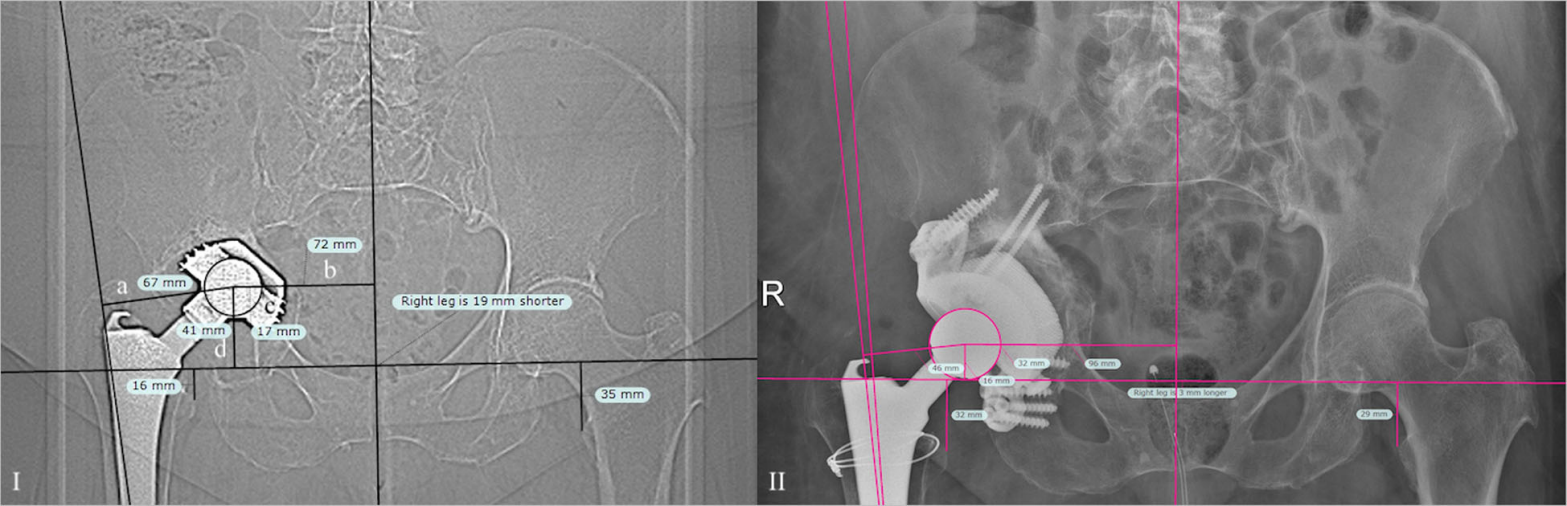
I) Measurement of offsets in a hip with aseptic loosening (**a**: femoral offset; **b**: medial offset; **c**: ilioischial offset; **d**: center of rotation) II) shows the same hip after revision with CTAC

Postoperative radiologic follow-up as shown in Fig. 5 showed no significant difference between medial and ilioischial offsets and height of COR in either hip. However, a significant difference in femoral offsets was noticed (Table 3). The median anteversion and inclination angles were 17 (range 6–25) and 45 (36–67) degrees, respectively (Table 3).

**Table 3:** Clinical and radiological results

	aMace (n = 9)		
Anteversion (deg)	17 (6–25)		
Inclination (deg)	45 (36–67)		
	preoperative	postoperative	p
Oxford hip score (48)	18 (11–43)	30 (16–47)	0.009
Harris hip score (100)	53 (23–92)	77 (46–92)	0.010
	Contralateral side	postoperative	p
Femoral offset (mm)	40 (31–54)	34 (24–49)	0.044
Medial offset (mm)	89 (78–103)	96 (83–120)	0.109
Ilioischial offset (mm)	27 (23–34)	30 (12–42)	0.568
Center of rotation (mm)	17 (7–31)	19 (3–38)	0.980

Data are presented as the median (range)

### Complications

Intraoperative microbial samples were positive in one patient who received intravenous antibiotics thereafter. One patient had sciatic nerve palsy due to the intervention. Three of the patients faced postinterventional complications; the patient with sciatic nerve palsy also developed deep vein thrombosis, and two other patients experienced femoroacetabular dislocation. Complications occurred in three over nine patients, resulting in a complication rate of 33.3%. One of these patients underwent a subsequent surgery for changing the type of inlay due to multiple dislocations (re-revision rate, 11.1%). This intervention led to stable conditions of the hip, and no further dislocations were noticed.

## Discussion

The current study evaluated the functional and radiological outcomes after the use of CTACs in patients with severe acetabular bone defects. Overall, satisfying clinical and radiological outcomes were observed. The OHS and HHS improved significantly one year postoperatively. Our results are comparable to recent research results. In a study by Wind et al., the authors investigated 19 patients after an average follow-up of 31 months after the use of CTACs. The authors found a significantly improved HHS from 38 to 63 points [22]. In another study by Taunton et al., the investigation of 57 patients with pelvic discontinuity at an average of 65 months after the use of CTACs showed a final HHS of 74.8 points [23]. With an average of 10 years, the study with the longest follow-up, performed by DeBoer et al., reported an improvement in the HHS from 41 to 80 points [24]. A recent published systematic review by Chiarlone et al. investigated acetabular custom-made implants for severe acetabular bone defect and showed satisfactory clinical and radiological outcomes at mid-term follow-up [25].

Radiological measurements of the COR and offsets showed no significant difference when compared to the “healthy” side except femoral offset (*p* = 0.044). However, most of the patients had already received THA on the contralateral side. The current measurements therefore should not be seen as a comparison to baseline data; rather, it shows the correct position of the aMace implant compared to the contralateral hip, regardless of preexisting THA. The significant difference in femoral offset (*p* = 0.044) is most likely due to an increase in medial offset and thus, compensation of global offset with an decrease of femoral offset. However, restoration of the center of rotation is important for postoperative improvement and stability after THA. We achieved this in eight of nine patients with a mean COR of 19.4 mm. A “high hip center of rotation” is defined in the literature with a COR of more than 35 mm [12, 26]. Only one patient did not match the limit with a distance to the inter-teardrop line of 38 mm. Durand-Hill et al. reported a 100% rate of correct implant position as planned preoperatively [27]. However, they did not investigate the position compared to the contralateral side. Others reported satisfying final position of the implants, inclination of the shell or osseointegration, but did not investigate offsets and COR [28, 29]. A recently published study by Walter et al. showed significant lateralisation and cranialization when CTAC was used [30]. This study cannot confirm these findings as shown above.

One patient faced postoperative dislocation of the hip 87 days after implantation of the aMace cup. This special case may have been due to too little anteversion, which was 6 degrees in that particular patient. The patient received revision surgery with implantation of a dual mobility cup. No further dislocation was noticed thereafter. None of the other patients experienced postoperative dislocation; in these cases, the anteversion was at a mean of 16.1 degrees.

A review of up-to-date literature shows complications in 16 to 53% of patients and re-revision in 11 to 35% [24, 25, 31–34]. We experienced a complication rate of 33.3%, resulting in a re-revision rate of 11.1%, which matched the rates in these trials. None of the implants had to be removed due to postoperative complications.

During surgery, less screws were used to fix the implant (13.7 planned, 9 used). This is mainly due to intersection of some screws, where the surgeon has to select the preferred screw preoperatively. In some additional cases, a screw could not be positioned due to the surgical approach.

Of course, this study has several limitations. The main limitations are the small sample size and the lack of a control group as well as its retrospective design. To achieve a true statement about restoration of COR and offsets, it would need a patient population without arthroplasty on the healthy side. Only retrospective trials are investigating custom made hip cups for severe acetabular defects are available. However, prospectively planned trial would be favourable and should be conducted in the future.

Finally, implant cost is a factor that should be taken into consideration during the decision process. While planning is free of charge, the set of one Materialise aMace implant plus drilling guides and 3D models costs approximately 15.000€. In the authors’ opinion, the implant is a reasonable alternative for patients with severe acetabular bone defects. However, long-term outcomes still need to be examined, and cost-benefit analysis should be conducted to investigate a potential long-term benefit.

## Conclusion

Revision surgery in THA with Materialise aMace custom-made acetabular components in patients with severe acetabular bone loss shows promising short-term functional and radiological outcomes. However, long-term outcomes still need to be examined.

## Data Availability

This manuscript does not contain any individual person’s data in any form.

## Abbreviations

COR: Center of rotation
CRP: C-reactive protein
CT: Computed tomography
CTAC: Custom-made triflanged acetabular components
HHS: Harris Hip Score
IBG: Impaction bone grafting
OHS: Oxford Hip Score
SD: Standard deviation
THA: Total hip arthroplasty
WBC: White blood count
